# Understanding the Public’s Emotions about Cancer: Analysis of Social Media Data

**DOI:** 10.3390/ijerph17197160

**Published:** 2020-09-30

**Authors:** Seul Ki Park, Hyeoun-Ae Park, Jooyun Lee

**Affiliations:** 1 College of Nursing and Research Institute of Nursing Science, Seoul National University, Seoul 03080, Korea; gomtty3@snu.ac.kr; 2 College of Nursing, Gachon University, Incheon 21936, Korea; jooyun@gachon.ac.kr

**Keywords:** social media, emotional analysis, cancer, association rule mining, social network analysis

## Abstract

Cancer survivors suffer from emotional distress, which varies depending on several factors. However, existing emotion management programs are insufficient and do not take into consideration all of the factors. Social media provides a platform for understanding the emotions of the public. The aim of this study was to explore the relationship between the public’s emotions about cancer and factors affecting emotions using social media data. We used 321,339 posts on cancer and emotions relating to cancer extracted from 22 social media channels between 1 January 2014, and 30 June 2017. The factors affecting emotions were analyzed using association rule mining and social network analysis. Hope/gratitude was the most frequently mentioned emotion group on social media followed by fear/anxiety/overwhelmed, sadness/depression/loneliness/guilt, and anger/denial. Acute survival stage, treatment method, and breast cancer were associated with hope/gratitude. Early stage, gastrointestinal problems, fatigue/pain/fever, and pancreatic cancer were associated with fear/anxiety/overwhelmed. Surgery, hair loss/skin problems, and fatigue/pain/fever were associated with sadness/depression/loneliness/guilt. Acute survival stage and hair loss/skin problems were associated with anger/denial. We found that emotions concerning cancer differed depending on the cancer type, cancer stage, survival stage, treatment, and symptoms. These findings could guide the development of tailored emotional management programs for cancer survivors that meet the public’s needs more effectively.

## 1. Introduction

Cancer is a significant disease burden worldwide. In Korea, cancer-related deaths accounted for 26.5% of all deaths in 2018, and cancer has been ranked as the leading cause of death since 1983 [1]. However, with the recent advances in cancer prevention, early detection, treatment, and follow-up care, the five-year survival rate for all cancers combined improved from 44.0% during 1996–2000 to 70.4% during 2013–2017 in Korea [2]. Since the life expectancy of cancer patients has increased, cancer survivors may face physical and emotional hardships from cancer-related and treatment-related short-term, long-term, and late health effects. Cancer survivors experience various physical challenges, such as physical impairment, severe fatigue, vomiting, and loss of appetite. They also face numerous emotional problems, including anger, fear, anxiety, depression, and loneliness, throughout the cancer trajectory. Many cancer survivors suffer from severe emotional distress, which can impair their ability to cope with cancer effectively and impact their quality of life. Anxiety and depression, which are most common among cancer survivors, have been reported to result in poor adherence to cancer treatment, poor cancer survival, and increased risk of suicide if not properly managed [3,4]. Emotional distress in cancer survivors has been associated with several patient and disease characteristics, such as age [5], gender [6], cancer type [7], and stage [7,8]. Hence, cancer survivors are increasingly interested in how to deal with these chronic emotional challenges. The general public also has an increasing need for information concerning cancer-related emotion because they may have cancer survivors around them as family members, friends, or co-workers [9].

The National Cancer Institute and the American Cancer Society (United States), as well as Cancer Research UK (UK), provide comprehensive cancer information on their websites. They also provide information on emotional problems experienced by cancer survivors, as well as providing information regarding the management of emotions. However, the emotions experienced by cancer survivors may vary depending on the gender, age, cancer type, and cancer stage. Thus, it is necessary to provide tailored information on emotional management, which requires a deep understanding of the emotions experienced by the public regarding cancer. Recently, the use of data obtained from social media has been proposed as a novel approach to understand the public’s emotional reaction to it. Social media platforms, such as social networking services (SNS), internet blogs, and online communities, have gained increasing popularity with the spread of smartphones and the internet over the last decade. Through these platforms, individuals report personal experiences, opinions, and emotions about cancer, sharing them with others in real-time [10]. Social media posts can be very useful in understanding the public’s emotions relating to cancer. For example, Crannell et al. [11] analyzed 146,357 tweets from cancer survivors and found that patients with cancer with favorable prognoses had higher happiness scores than patients with cancers with poor prognoses. However, this and other similar studies using social media data were limited to only one emotion [11] or classified emotions only as positive, negative, or neutral [12,13,14,15]. Thus, these studies did not provide a comprehensive insight into the public’s various emotions relating to cancer, such as depression, anxiety, hope, and anger. Additionally, social media data were collected in the previous research using only a few keywords for each topic of interest, which might not be sufficient to capture the full scope of emotions relating to cancer. As social media posts are written in colloquial language, consumer terms must be used to collect social media data. Thus, a list of concepts and terms for emotions about cancer used by the consumers provides a more appropriate framework for the collection and analysis of such data.

In this study, we used a cancer ontology previously developed by the authors [16] as a framework for social media data collection and analysis to investigate the public’s emotions concerning cancer. The ontology contained nine superclasses (cancer type, prevention, diagnosis, treatment, prognosis, risk factor, symptom, dealing with cancer, and emotion), 213 class concepts, and 4061 synonyms. The emotion superclass, which was one of the nine superclasses, was composed of nine classes (denial, anger, overwhelmed, anxiety, depression, loneliness, guilt, hope, and gratitude) with 454 synonyms. The synonyms included colloquial expressions (heteronyms, abbreviations, and slang) used by the public in daily life, which are useful for collecting social media data. This study explores the relationship between the public’s emotions about cancer and the factors affecting these emotions by association rule mining and social network analysis. The relationship between the public’s emotions relating to cancer and the factors affecting these emotions identified in this study will provide a framework for establishing tailored health information that supports the management of emotions. 

## 2. Materials and Methods 

In this study, we used 321,339 posts on cancer and cancer-related emotions. These posts were obtained from online cafés (online communities) operated by Naver and Daum, internet blogs operated by Naver, Daum, Tistory, and Egloos, Twitter, and 15 message boards (e.g., YouTube, Naver Knowledge iN, Nate talk), between 1 January 2014, and 30 June 2017, in Korea. Naver and Daum are the two largest online platforms in Korea. Tistory and Egloos are blogging platforms in Korea.

### 2.1. Data Collection 

We collected posts on cancer and cancer-related emotions. As search keywords, we used 302 terms for concepts and synonyms of the cancer type superclass (such as malignant cancer, colon cancer, brain cancer, liver cancer, and breast cancer) and 454 terms for concepts and synonyms of the emotion superclass (such as surprise, embarrassment, contempt, anger, and worry) contained in the cancer ontology developed by the authors. As stop keywords, we used 418 terms (such as malicious virus). We collected posts on cancer, from which we then selected posts on emotions relating to cancer.

Of the 1,854,497 posts on cancer, 434,299 posts included keywords indicating at least one emotion. Of these, 112,960 posts contained advertising keywords (such as detoxification) and were excluded. Finally, a total of 321,339 posts were selected for analysis. Terms were extracted from the posts using ontology-based natural language processing (NLP). The data collection and NLP procedures were performed in collaboration with SK Telecom Smart Insight, a Korea-based big-data marketing platform. The company used web crawler and Java-based NLP tools, which were developed within the company. Each document was word tokenized and mentions of emotions and factors related to emotions in each document were identified using terms in cancer ontology.

The study was approved by the Institutional Review Board of the Seoul National University (IRB No. 1802/001-006). Collected posts did not have any identifiable personal information. 

### 2.2. Data Preparation

The collected social media data were converted into structured data for analysis. A single post was treated as an analysis unit. After extracting terms from each post, we identified terms for emotions and factors affecting the emotions, which were included in the cancer ontology. 

Emotions concerning cancer were defined as per the emotion classification proposed by Jack et al. [17] as “Happy”, “Surprise/Fear”, “Sad”, and “Disgust/Anger”. The nine emotions included in the cancer ontology were divided into four emotion groups as per the classification by Jack et al. as “Hope/Gratitude”, “Fear/Anxiety/Overwhelmed”, “Sadness/Depression/Loneliness/Guilt”, and “Anger/Denial”. We coded the posts based on the presence of terms indicating each of four emotion groups as 0 (=no) or 1 (=yes). If more than two emotion groups were mentioned in a single post, each emotion group was counted. If the same emotion group was mentioned multiple times in a single post, it was only counted once.

Factors affecting emotions, such as gender, age, cancer type, stage, treatment, survival stage, and symptoms were defined. Gender was defined as male and female, and individuals were grouped according to age as <10, 10 s, 20 s, 30 s, 40 s, 50 s, 60 s, 70 s, and >80. Cancer types were divided into 14 groups, namely breast cancer, colon cancer, gastric cancer, leukemia, lung cancer, cervical cancer, liver cancer, brain cancer, pancreatic cancer, ovarian cancer, prostatic cancer, gallbladder cancer, kidney cancer, and thyroid cancer, by combining the top 10 cancers in national cancer statistics in Korea, 2016 [18] and social media posts. The cancer stage was defined as early stage, middle stage, and terminal stage. Treatments were classified as surgery, chemotherapy, radiation therapy, immunotherapy, complementary and alternative medicine, and transplantation. Survival stages were classified as acute survival stage, extended survival stage, and permanent survival stage. Symptoms were classified as general symptoms of fatigue/pain/fever, gastrointestinal problems, skin problems, poor circulation, thrombocytopenia, and infection. We coded the posts based on the presence of terms indicating each of the 43 emotion-related factor groups as 0 (=no) or 1 (=yes). If more than two emotion-related factor groups were mentioned in one post, each factor group was counted. If the same emotion-related factor group was mentioned multiple times in a single post, it was only counted once.

### 2.3. Data Analysis

#### 2.3.1. Frequency Analysis of Post

We analyzed the frequency of the general characteristics mentioned in the post of each social media channel. We also analyzed the frequency of the four emotion groups for each social media channel.

#### 2.3.2. Association Rule Mining

We performed emotional analysis using association rule mining by applying the Apriori algorithm to investigate the relationship between different emotion-related factors and emotion groups. Three main measures of the association rule mining were support, confidence, and lift. Support indicated the proportion of posts containing emotion-related factors and emotion groups in the entire posts. Confidence indicated the proportion of posts containing emotion groups among posts with emotion-related factors. Lift indicated the ratio of the appearance of an emotion group in posts with emotion-related factors to the appearance of that group in all posts. Rules with lift values >1 indicated a positive correlation, whereas rules with lift values <1 indicated a negative correlation. Lift values close to 1 indicated no association between emotion-related factors and emotion groups [19]. Association rule mining was performed using the R software package (version 3.6.0).

#### 2.3.3. Social Network Analysis 

We classified social media according to the post’s length, as internet blogs, online cafés, and message boards allow for long posts, whereas Twitter posts are limited to 140 characters. We performed social network analysis to examine and visualize relationships between emotion groups or emotion-related factors that appeared together in each post in the two groups. The nodes in the network represent the emotion groups and emotion-related factors, while the edges indicate relationships between the nodes. Node activity was determined based on the degrees corresponding to the number of direct connections to the node. The strength of the relationship between nodes was assessed based on the edge weights corresponding to the number of interactions [20]. The network of the relationships between emotion groups and emotion-related factors was constructed using NetMiner 4.4.3.b (Cyram Inc., Seoul, Korea).

## 3. Results

### 3.1. Frequency Analysis of Posts

From the total of 321,339 posts analyzed, 128,944 posts (40.1%) were from internet blogs, 125,976 posts (39.2%) were from online cafés, 61,180 posts (19.0%) were from Twitter, and 5239 posts (1.6%) were from message boards.

#### 3.1.1. Frequency of General Characteristics on Social Media Channels

We analyzed the frequencies of terms describing gender, age, and cancer type mentioned on social media channels (Table 1). Both genders were most commonly mentioned in online cafés, followed by internet blogs, Twitter, and message boards. Although age groups <60 were most commonly mentioned on internet blogs, age groups over 60 were most commonly mentioned in online cafés. Age groups <60 were mentioned in 2.3–9.7% of Twitter posts; however, ages over 60 were mentioned in only 0.6–0.7% of Twitter posts. All cancer types, except leukemia, were most frequently mentioned on internet blogs and online cafés. Notably, gallbladder cancer was mentioned twice as much in online cafés (65.1%) than on internet blogs (33.4%). Leukemia was the most frequently mentioned cancer type on Twitter.

#### 3.1.2. Frequency of Emotion Groups

Hope/gratitude was the most frequently mentioned emotion group on social media (*n* = 149,558; 46.5%), followed by fear/anxiety/overwhelmed (*n* = 145,179, 45.2%), sadness/depression/loneliness/guilt (*n* = 126,496, 39.4%), and anger/denial (*n* = 25,560; 8.0%).

### 3.2. Association Rule Mining

The relationship between emotion groups and emotion-related factors was assessed using association rule mining. The top five association rules with the highest lift are shown in Table 2.

#### 3.2.1. Hope/Gratitude

“Radiation therapy”, “Acute survival stage”, “Male”, and “Female” were identified to be strongly associated with hope/gratitude (support, 0.010; confidence, 0.911; lift, 1.957). The support level of 0.010 indicated that the proportion of posts containing “Radiation therapy”, “Acute survival stage”, “Male”, “Female”, and hope/gratitude among total posts was 0.010. The confidence level of 0.911 indicated that the proportion of posts with hope/gratitude emotions among the posts mentioning “Radiation therapy”, “Acute survival stage”, “Male”, and “Female” was 0.911. The lift level of 1.957 indicated that the ratio of the appearance of hope/gratitude in posts mentioning “Radiation therapy”, “Acute survival stage”, “Male”, and “Female” to the appearance of hope/gratitude in total posts was 1.957. “Chemotherapy”, “Breast cancer”, “Surgery”, and “Liver cancer” were also strongly associated with hope/gratitude, with a lift value of >1.89.

#### 3.2.2. Fear/Anxiety/Overwhelmed

“Early stage” and “Gastrointestinal problems” were associated with fear/anxiety/overwhelmed, with a support value of 0.016, a confidence value of 0.773, and a lift value of 1.623. “Chemotherapy”, “Fatigue/Pain/Fever”, “Pancreatic cancer”, “Acute survival stage”, and “Brain cancer” were also associated with fear/anxiety/overwhelmed, with a lift value of >1.61.

#### 3.2.3. Sadness/Depression/Loneliness/Guilt

“Hair loss/Skin problems”, “Male”, and “Female” were associated with sadness/depression/loneliness/guilt, with a support value of 0.014, a confidence value of 0.767, and a lift value of 1.949. “Surgery” and “Fatigue/Pain/Fever” were also identified as factors strongly associated with sadness/depression/loneliness/guilt, with a lift value of >1.77.

#### 3.2.4. Anger/Denial

“Hair loss/Skin problems” was associated with anger/denial, with support, confidence, and lift values of 0.010, 0.129, and 1.619, respectively. “Acute survival stage”, “Male”, and “Female” were also strongly associated with anger/denial (lift value >1.35). The factors associated with anger/denial showed a very low confidence value (0.107–0.129) compared with those associated with the other three emotion groups (0.699–0.911).

### 3.3. Social Network Analysis

**Figure 1:**
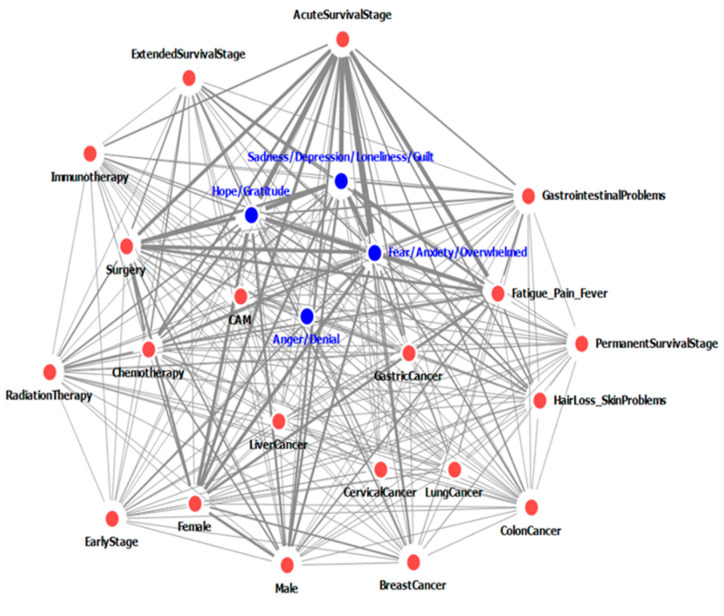
Network of emotion groups concerning cancer and factors affecting emotions groups, as determined through posts on internet blogs, online cafés, and message boards.

**Figure 2:**
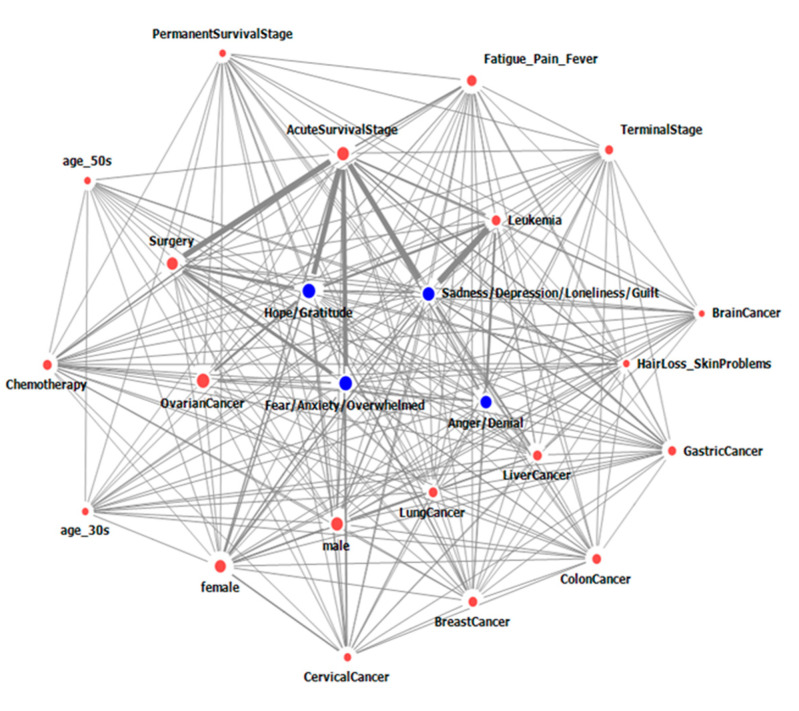
Network of emotion groups relating to cancer and factors affecting emotions groups, as determined through posts on Twitter.

Table 3 and Table 4, and Figure 1 and Figure 2 show the network of the top 24 nodes (50% of the total nodes) that represent emotion groups and factors related to emotion groups, which were mentioned on social media. Table 3 and Table 4 show the frequency, degree, and edge weight of nodes on internet blogs, online cafés, and message boards (Table 3) and Twitter (Table 4). Figure 1 and Figure 2 display the network of the relationship between nodes, including the degree and edge weight of the nodes in posts from internet blogs, online cafés, and message boards (Figure 1) and Twitter (Figure 2). The size of the nodes is proportional to the degree, and the thickness of the edges is proportional to the edge weight.

On internet blogs, online cafés, and message boards, the node representing “Fear/Anxiety/Overwhelmed” was the most frequent, followed by “Acute survival stage”, “Hope/Gratitude”, “Sadness/Depression/Loneliness/Guilt”, and “Fatigue/Pain/Fever”. Each of the 47 nodes was connected to the remaining 46 nodes. The two nodes with the highest edge weight were “Acute survival stage” and “Fear/Anxiety/Overwhelmed”, followed by “Acute survival stage—Hope/Gratitude”, “Acute survival stage—Sadness/Depression/Loneliness/Guilt”, and “Acute survival stage—Fatigue/Pain/Fever”. The two emotion groups with the highest edge weight were “Hope/Gratitude” and “Sadness/Depression/Loneliness/Guilt”, followed by “Sadness/Depression/Loneliness/Guilt—Fear/Anxiety/Overwhelmed” and “Hope/Gratitude—Fear/Anxiety/Overwhelmed” (Table 3, Figure 1).

On Twitter, the node with the highest frequency was “Hope/Gratitude”, followed by “Sadness/Depression/Loneliness/Guilt”, “Acute survival stage”, “Leukemia”, “Fear/Anxiety/Overwhelmed”, and “Anger/Denial”. “Hope/Gratitude”, “Fear/Anxiety/Overwhelmed”, “Acute survival stage”, “Sadness/Depression/Loneliness/Guilt”, and “Anger/Denial” were connected to more than 40 nodes. The two nodes with the highest edge weight were those representing “Leukemia” and “Sadness/Depression/Loneliness/Guilt”, followed by those representing “Acute survival stage–Hope/Gratitude”, “Acute survival stage—Sadness/Depression/Loneliness/Guilt”, “Acute survival stage—Surgery”, and “Acute survival stage—Fear/Anxiety/Overwhelmed” (Table 4, Figure 2).

## 4. Discussion

In this study, we explored the relationship between the public’s emotions about cancer and emotion-related factors by association rule mining and social network analysis of social media data based on a cancer ontology.

A frequency analysis of the public’s emotion for cancer revealed that hope/gratitude, the only positive emotion group, was the most common emotion group mentioned on social media, appearing in 46.5% of all posts. A previous study calculating the happiness value of cancer patients and the general public on Twitter [11] found that the computed happiness value was higher for cancer patients’ tweets than for those from the general public and that negative words were less frequent among the tweets of cancer patients. Cancer survivors may be more thankful for being cancer-free or grateful and appreciative of their family. Moreover, cancer survivors not only record their experiences and feelings but also read other people’s posts and provide positive emotional support and encouragement to each other, according to the study by Lieberman and Goldstein [21]. Hope and gratitude have a beneficial effect on physical health, psychological wellbeing, and the quality of life of cancer survivors [22].

Fear/anxiety/overwhelmed and sadness/depression/loneliness/guilt appeared in 45.2% and 39.4% of the total posts, respectively, consistent with a previously reported prevalence of anxiety and depression among cancer patients and caregivers [6,23]. Linden et al. [6] reported that the frequency of anxiety symptoms was 41.6% in 10,153 Canadian cancer patients. Similarly, in a meta-analysis involving 21,149 caregivers, Geng et al. [23] found that the frequencies of anxiety and depression were 46.55% and 41.0%, respectively. Uncertainty of the progress and prognosis of cancer could have contributed to the high prevalence of anxiety in cancer patients and caregivers. Herschbach et al. [24] found that the most important psychological distress in cancer patients was anxiety and fear. Notably, cancer patients were afraid of disease progression, re-hospitalization, pain, and not being fit for work. Sadness and depression are also prevalent feelings among cancer patients and caregivers. Although several studies have reported that depression in cancer patients and caregivers impacts their quality of life, treatment compliance, subjective perception of physical symptoms, and prognosis [3,4], its diagnosis is often overlooked by healthcare providers. Thus, it is crucial that healthcare providers diagnose anxiety and depression in cancer patients and caregivers.

Anger/denial was the less frequently mentioned emotion group on social media, with a frequency of 8.0%. Consistently, Hadi et al. [25] found that anger was less frequent in breast cancer patients than were depression and anxiety. They also found that the anger score in breast cancer patients was significantly lower than that in the general public. Our study and Hadi et al.’s study suggest that cancer survivors may suppress or restrain the expression of anger, potentially leading to distress and depression [26], although anger or denial is the first emotion that most patients experience after cancer diagnosis [27]. Since anger suppression is known to have a negative effect on cancer prognosis [28], the development of interventions that manage anger in cancer patients is urgently needed.

In this study, we also identified factors associated with each emotion group using association rules mining. Acute survival stage, breast cancer, and treatment methods, including radiation therapy, chemotherapy, and surgery, were associated with hope/gratitude. Among those factors, acute survival stage was also associated with anger/denial. According to Mullan [29], acute survival stage is the first stage of cancer survivorship and includes the time of diagnosis until the initial treatment, such as surgery or radiotherapy. After being diagnosed with cancer, many people experience denial and anger. Moreover, people at this stage need practical assistance, such as medical information or social support, and healthcare providers and family members usually give hopeful messages about the prognosis after treatment. Breast cancer was also associated with hope/gratitude, which is likely to be because it is the most common cancer among Korean women and has the second-highest survival rate after thyroid cancer [30]. Breast cancer online cafés have the highest number of members among all cancer-related online cafés in Korea. Breast cancer patients often share health information on their treatment, symptoms, and emotional support. In contrast to men, women tend to share feeling-centered and emotion-focused supportive messages [31].

Early stage disease, gastrointestinal problems, fatigue/pain/fever, and pancreatic cancer were factors associated with fear/anxiety/overwhelmed. Patients with early stage cancer have a considerably better prognosis [32]. However, physical side effects, such as pain, fever, or gastrointestinal symptoms, result in anxiety and fear. Even if the early stage tumor is completely removed during surgery, the possibility of residual disease causes fear and anxiety [33]. Pancreatic cancer was associated with fear/anxiety/overwhelmed. Consistently, Zabora et al. [7] reported that pancreatic cancer had the highest anxiety score among 14 cancer types. Additionally, pancreatic cancer has an extremely poor prognosis, with a 5-year survival rate of only 12% in Korea [34]. Therefore, people with these factors require special attention regarding fear and anxiety.

Surgery, hair loss/skin problems, and fatigue/pain/fever were associated with sadness/depression/loneliness/guilt. Among these factors, hair loss/skin problems were also associated with anger/denial. Surgery is one of the most common treatments for cancer. Cancer patients often experience depression before surgery due to uncertainty about the surgery outcome, anesthesia, death, fear of postoperative pain, or complications. They also feel depressed after surgery due to pain and physical changes. Patients who experience loss of body parts, such as those undergoing mastectomy [35] or colostomy [36], often experience severe depression. Hair loss is a common side effect of chemotherapy [37]. Although hair loss is not permanent or life-threatening, it has a tremendous psychological impact on patients, as it is a drastic change in physical appearance. Fatigue is a common treatment-related physical symptom, experienced by 90% of cancer patients receiving radiation therapy and 100% of those receiving chemotherapy [38]. Although most healthy people recover from fatigue with sleep and rest, cancer patients suffer from depression and chronic fatigue due to their disease and the side effects of the treatment [39].

An internet blog is a personal platform where individuals can write about their interests, whereas online cafés are communities where people share common interests. In this study, we found that gallbladder cancer and pancreatic cancer, both of which have a poor prognosis, were mentioned more frequently in online cafés than on internet blogs, perhaps due to a potential preference to share their experiences with many people and acquire emotional comfort through an online café. In this study, we found that younger people (10–50 years old) were most frequently mentioned on internet blogs, whereas older people (>60 years old) were most frequently mentioned in online cafés. Younger people are more familiar with social media and often communicate through their own internet blogs. In contrast, older people tend to communicate through established online communities [40]. In terms of emotions and emotion-related factors, acute survival stage, fear/anxiety/overwhelmed, hope/gratitude, and sadness/depression/loneliness/guilt, were most commonly mentioned on internet blogs, online cafés, and message boards. This finding suggests that the general public is most active on social media for seeking information and emotional support during the acute survival stage and that various emotions, including hope, sadness, and fear, are mixed at this stage.

Twitter is a microblog, where users can post short messages to their followers. Since most Twitter users are aged between 10 and 30 [40], cancer types that are more common in young individuals, such as leukemia [41], were more frequently mentioned on Twitter than on internet blogs or online cafés. Leukemia patients or their parents who are the primary caregivers post about the disease and seek emotional support on Twitter. Moreover, leukemia and sadness/depression/loneliness/guilt were most frequently mentioned on Twitter. This could be because the parents, who are the principal caregivers of leukemia patients, feel guilty about their children. This hypothesis is supported by a study that showed that the most common emotion in mothers caring for their children with blood cancer was guilt [42]. Hence, tailored emotion management programs should be established for cancer patients of different ages and with different cancer types, but also for caregivers, considering social media usage patterns.

This study had some limitations. First, although various SNS are available in Korea, such as Kakaostory, Facebook, and Instagram [40], we were only able to collect data from Twitter because the data is the only publicly available on SNS. However, future studies are required to analyze data from other social media platforms. Second, the emotions expressed in the post may not be the emotions felt by the post writers, and the factors related to emotions including demographic factor may not be the writer’s actual information, so care must be taken when interpreting the data. Third, if several emotions were mentioned in a single post, the factors related to emotions may have been analyzed as factors related to various emotions. It is necessary to explore the context of the posts to understand which factors are related to each emotion. Fourth, although cancer incidence increases rapidly after the age of 65, social media is mainly used by young people. Hence, older people’s emotions in relation to cancer may have been under-represented in this study. It is necessary to collect data from other sources to understand the emotions of older people more fully.

## 5. Conclusions

We explored the relationship between the public’s emotions about cancer and the factors related to these emotions using social media data. By using consumer terms in the cancer ontology, we could collect comprehensive social media data related to cancer written by the consumers. We found that the most frequently mentioned emotion group was hope/gratitude, and general public engages in active social media activities during the acute survival stage, when they feel various emotions. Thus, it is especially important to manage the emotions of cancer survivors in the acute survival stage. Additionally, the usage patterns of social media channels differed depending on the age and cancer type. Younger generations or leukemia were mentioned more in social media pages, such as internet blogs and Twitter threads, created by affected individuals, whereas older people or gallbladder cancer were mentioned more in social media pages created by others, such as online cafés. Thus, internet blogs or Twitter could be used for younger people and online cafés could be used for older people as the primary social media channel for managing the emotions of cancer survivors. The findings of this study could guide the development of tailored emotion management programs for cancer survivors reflecting the general public’s needs by age, cancer type and stage, treatment type, symptoms, and survival stage.

## Figures and Tables

**Table 1:** Frequency of general characteristics on social media channels.

Characteristics	Total	Internet Blogs	Online Cafés	Twitter	Message Boards
**Gender, *n* (%)**					
Male	54,553 (100%)	20,755 (38.0%)	9939 (54.9%)	2717 (5.0%)	1142 (2.1%)
Female	67,044 (100%)	29,494 (44.0%)	32,925 (49.1%)	3464 (5.2%)	1161 (1.7%)
**Age, *n* (%)**					
Less than 10	8635 (100%)	4941 (57.2%)	3436 (39.8%)	200 (2.3%)	58 (0.7%)
10–19	6462 (100%)	3300 (51.1%)	2749 (42.5%)	168 (2.6%)	245 (3.8%
20s	4920 (100%)	2439 (49.6%)	1955 (39.7%)	277 (5.6%)	249 (5.1%)
30s	5007 (100%)	2587 (51.7%)	1920 (38.3%)	439 (8.8%)	61 (1.2%)
40s	5621 (100%)	2991 (53.2%)	2203 (39.2%)	378 (6.7%)	49 (0.9%)
50s	5740 (100%)	2840 (49.5%)	2298 (40.0%)	557 (9.7%)	45 (0.8%)
60s	872 (100%)	264 (30.3%)	595 (68.2%)	6 (0.7%)	7 (0.8%)
70s	633 (100%)	264 (41.7%)	357 (56.4%)	4 (0.6%)	8 (1.3%)
Over 80	601 (100%)	277 (46.1%)	313 (52.1%)	4 (0.7%)	7 (1.2%)
**Cancer type, *n* (%)**					
Breast cancer	27,085 (100%)	12,551 (46.3%)	12,363 (45.6%)	2046 (7.6%)	125 (0.5%)
Colon cancer	24,569 (100%)	11,387 (46.3%)	12,253 (49.9%)	773 (3.1%)	156 (0.6%)
Gastric cancer	21,470 (100%)	8812 (41.0%)	10,975 (51.1%)	1569 (7.3%)	114 (0.5%)
Leukemia	21,461 (100%)	5450 (25.4%)	6053 (28.2%)	9802 (45.7%)	156 (0.7%)
Lung cancer	19,442 (100%)	7919 (40.7%)	9549 (49.1%)	1798 (9.2%)	176 (0.9%)
Cervical cancer	18,617 (100%)	9342 (50.2%)	7609 (40.9%)	1518 (8.2%)	148 (0.8%)
Liver cancer	15,715 (100%)	5909 (37.6%)	8128 (51.7%)	1549 (9.9%)	129 (0.8%)
Brain cancer	14,176 (100%)	5571 (39.3%)	6824 (48.1%)	1648 (11.6%)	133 (0.9%)
Pancreatic cancer	10,627 (100%)	4461 (42.0%)	5800 (54.6%)	275 (2.6%)	91 (0.9%)
Ovarian cancer	9855 (100%)	3374 (34.2%)	4471 (45.4%)	1975 (20.0%)	35 (0.4%)
Prostatic cancer	7507 (100%)	4060 (54.1%)	3348 (44.6%)	57 (0.8%)	42 (0.6%)
Gallbladder cancer	3740 (100%)	1251 (33.4%)	2436 (65.1%)	32 (0.9%)	21 (0.6%)
Kidney cancer	2585 (100%)	1318 (51.0%)	1221 (47.2%)	22 (0.9%)	24 (0.9%)
Thyroid cancer	726 (100%)	308 (42.4%)	216 (29.8%)	197 (27.1%)	5 (0.7%)

**Table 2:** Association rules with top five lift by emotions.

Rules	Support	Confidence	Lift
***Hope/Gratitude***			
{Radiation therapy, Acute survival stage, Male, Female} => {Hope/Gratitude}	0.010	0.911	1.957
{Chemotherapy, Breast cancer, Male} => {Hope/Gratitude}	0.011	0.894	1.921
{Surgery, Liver cancer, Female} => {Hope/Gratitude}	0.010	0.890	1.913
{Surgery, Chemotherapy, Acute survival stage, Male, Female} => {Hope/Gratitude}	0.010	0.887	1.906
{Radiation therapy, Chemotherapy, Male, Female} => {Hope/Gratitude}	0.010	0.883	1.897
***Fear/Anxiety/Overwhelmed***			
{Early stage, Gastrointestinal problems} => {Fear/Anxiety/Overwhelmed}	0.016	0.733	1.623
{Early stage, Chemotherapy} => {Fear/Anxiety/Overwhelmed}	0.015	0.732	1.620
{Fatigue/Pain/Fever, Pancreatic cancer} => {Fear/Anxiety/Overwhelmed}	0.011	0.731	1.619
{Early stage, Acute survival stage, Fatigue/Pain/Fever} => {Fear/Anxiety/Overwhelmed}	0.013	0.731	1.618
{Acute survival stage, Fatigue/Pain/Fever, Brain cancer} =>{Fear/Anxiety/Overwhelmed}	0.011	0.731	1.617
***Sadness/Depression/Loneliness/Guilt***			
{Hair loss/Skin problems, Male, Female} => {Sadness/Depression/Loneliness/Guilt}	0.014	0.767	1.949
{Surgery, Fatigue/Pain/Fever, Male, Female} => {Sadness/Depression/Loneliness/Guilt}	0.010	0.742	1.886
{Surgery, Hair loss/Skin problems, Female} => {Sadness/Depression/Loneliness/Guilt}	0.010	0.731	1.857
{Hair loss/Skin problems, Male} => {Sadness/Depression/Loneliness/Guilt}	0.018	0.702	1.782
{Fatigue/Pain/Fever, Male, Female} => {Sadness/Depression/Loneliness/Guilt}	0.024	0.699	1.775
***Anger/Denial***			
{Hair loss/Skin problems} => {Anger/Denial}	0.010	0.129	1.619
{Male, Female} => {Anger/Denial}	0.012	0.129	1.618
{Acute survival stage, Male} => {Anger/Denial}	0.012	0.116	1.458
{Acute survival stage, Female} => {Anger/Denial}	0.014	0.109	1.376
{Male} => {Anger/Denial}	0.018	0.107	1.351

**Table 3:** Top 24 nodes with degree and edge weight on internet blogs, online cafés, and message boards.

Emotion Groups and Emotion-Related Factors	Frequency *n* (%)	Emotion Groups and Emotion-Related Factors	Degree	Emotion Groups and Emotion-Related Factors	Emotion Groups and Emotion-Related Factors	Edge Weight
Fear/Anxiety/Overwhelmed	135,986 (52.3)	Fear/Anxiety/Overwhelmed	46	Acute survival stage	Fear/Anxiety/Overwhelmed	77,049
Acute survival stage	132,362 (50.9)	Acute survival stage	46	Acute survival stage	Hope/Gratitude	63,078
Hope/Gratitude	126,639 (48.7)	Hope/Gratitude	46	Acute survival stage	Sadness/Depression/Loneliness/Guilt	55,949
Sadness/Depression/Loneliness/Guilt	103,646 (39.8)	Sadness/Depression/Loneliness/Guilt	46	Acute survival stage	Fatigue/Pain/Fever	49,137
Fatigue/Pain/Fever	68,582 (26.4)	Fatigue/Pain/Fever	46	Hope/Gratitude	Sadness/Depression/Loneliness/Guilt	45,090
Female	63,580 (24.4)	Female	46	Sadness/Depression/Loneliness/Guilt	Fear/Anxiety/Overwhelmed	44,452
Surgery	63,310 (24.3)	Surgery	46	Hope/Gratitude	Fear/Anxiety/Overwhelmed	44,212
Male	51,836 (19.9)	Male	46	Fear/Anxiety/Overwhelmed	Fatigue/Pain/Fever	42,938
Chemotherapy	47,276 (18.2)	Chemotherapy	46	Female	Acute survival stage	40,806
Gastrointestinal problems	31,455 (12.1)	Gastrointestinal problems	46	Surgery	Acute survival stage	40,303
Extended survival stage	29,381 (11.3)	Extended survival stage	46	Surgery	Fear/Anxiety/Overwhelmed	37,629
Breast cancer	25,039 (9.6)	Breast cancer	46	Female	Fear/Anxiety/Overwhelmed	36,658
Hair loss/Skin problems	24,982 (9.6)	Hair loss/Skin problems	46	Female	Hope/Gratitude	35,553
Colon cancer	23,796 (9.1)	Colon cancer	46	Hope/Gratitude	Fatigue/Pain/Fever	34,780
Radiation therapy	20,642 (7.9)	Radiation therapy	46	Surgery	Hope/Gratitude	34,192
Gastric cancer	19,901 (7.6)	Gastric cancer	46	Sadness/Depression/Loneliness/Guilt	Fatigue/Pain/Fever	33,431
Immunotherapy	19,075 (7.3)	Immunotherapy	46	Female	Sadness/Depression/Loneliness/Guilt	33,325
Early stage	18,709 (7.2)	Early stage	46	Male	Acute survival stage	31,778
Lung cancer	17,644 (6.8)	Lung cancer	46	Surgery	Sadness/Depression/Loneliness/Guilt	30,742
Anger/Denial	17,121 (6.6)	Anger/Denial	46	Male	Hope/Gratitude	30,115
Cervical cancer	17,099 (6.6)	Cervical cancer	46	Male	Fear/Anxiety/Overwhelmed	29,293
Permanent survival stage	15,686 (6.0)	Permanent survival stage	46	Male	Sadness/Depression/Loneliness/Guilt	29,274
CAM ^1^	14,795 (5.7)	CAM ^1^	46	Male	Female	28,833
Liver cancer	14,166 (5.4)	Liver cancer	46	Chemotherapy	Acute survival stage	28,613

^1^ Complementary and alternative medicine.

**Table 4:** Top 24 nodes with degree and edge weight on Twitter.

Emotion Groups and Emotion-Related Factors	Frequency *n* (%)	Emotion Groups and Emotion-Related Factors	Degree	Emotion Groups and Emotion-Related Factors	Emotion Groups and Emotion-Related Factors	Edge Weight
Hope/Gratitude	22,919 (37.5)	Hope/Gratitude	45	Leukemia	Sadness/Depression/Loneliness/Guilt	5399
Sadness/Depression/Loneliness/Guilt	22,850 (37.3)	Fear/Anxiety/Overwhelmed	45	Acute survival stage	Hope/Gratitude	5270
Acute survival stage	14,706 (24.0)	Male	43	Acute survival stage	Sadness/Depression/Loneliness/Guilt	4925
Leukemia	9802 (16.0)	Acute survival stage	43	Acute survival stage	Surgery	4630
Fear/Anxiety/Overwhelmed	9193 (15.0)	Sadness/Depression/Loneliness/Guilt	43	Acute survival stage	Fear/Anxiety/Overwhelmed	4113
Anger/Denial	8439 (13.8)	Female	41	Surgery	Fear/Anxiety/Overwhelmed	2387
Surgery	4657 (7.6)	Surgery	40	Leukemia	Anger/Denial	2160
Female	3464 (5.7)	Anger/Denial	40	Leukemia	Hope/Gratitude	2012
Male	2717 (4.4)	Fatigue/Pain/Fever	35	Ovarian cancer	Hope/Gratitude	1929
Breast cancer	2046 (3.3)	Liver cancer	34	Surgery	Hope/Gratitude	1785
Ovarian cancer	1975 (3.2)	Colon cancer	34	Female	Hope/Gratitude	1449
Lung cancer	1798 (2.9)	Lung cancer	34	Female	Acute survival stage	1301
Brain cancer	1648 (2.7)	Chemotherapy	34	Male	Sadness/Depression/Loneliness/Guilt	1140
Gastric cancer	1569 (2.6)	Leukemia	33	Acute survival stage	Hair loss/Skin problems	1122
Liver cancer	1549 (2.5)	Breast cancer	33	Liver cancer	Sadness/Depression/Loneliness/Guilt	1118
Cervical cancer	1518 (2.5)	Gastric cancer	32	Breast cancer	Hope/Gratitude	1085
Hair loss/Skin problems	1124 (1.8)	Terminal stage	32	Female	Sadness/Depression/Loneliness/Guilt	1066
Fatigue/Pain/Fever	954 (1.6)	Extended survival stage	31	Gastric cancer	Acute survival stage	1044
Chemotherapy	946 (1.5)	Cervical cancer	29	Cervical cancer	Acute survival stage	995
Colon cancer	773 (1.3)	Age 10–19	27	Acute survival stage	Anger/Denial	983
Age-50s	557 (0.9)	Age-20s	27	Male	Fear/Anxiety/Overwhelmed	944
Age-30s	439 (0.7)	Permanent survival stage	27	Chemotherapy	Acute survival stage	929
Permanent survival stage	416 (0.7)	Hair loss/Skin problems	27	Acute survival stage	Fatigue/Pain/Fever	927
Terminal stage	379 (0.6)	Brain cancer	26	Acute survival stage	Leukemia	900
